# Interface dosimetry for electronic brachytherapy intracavitary breast balloon applicators

**DOI:** 10.1120/jacmp.v12i2.3221

**Published:** 2011-03-02

**Authors:** James J. Segala, Gene A. Cardarelli, Jessica R. Hiatt, Bruce H. Curran, Edward S. Sternick

**Affiliations:** ^1^ Department of Radiation Oncology Rhode Island Hospital Providence Rhode Island 02903 USA

**Keywords:** accelerated partial breast irradiation, electronic brachytherapy, Monte Carlo simulation

## Abstract

In this study, we evaluate the attenuation of the dose due to barium‐impregnation in the region between the surface of an electronic brachytherapy (EBT) balloon applicator for accelerated partial breast irradiation (APBI) and the prescription point at 1 cm depth in tissue. To perform the study, depth dose curves were calculated using a general purpose multi‐particle transport code (FLUKA) for a range of balloon wall thicknesses with and without barium impregnation. Numerical data were verified with experimental readings using a parallel plate extrapolation ionization chamber for different wall thicknesses. Depth dose curves computed using both numerical and experimental methods show a 6.0% attenuation of the dose at the 1.0 cm prescription line due to the impregnation of barium in the balloon material, which agrees well with the manufacturer's specification. By applying this single attenuation factor, dose calculations throughout the entire planned volume are uniformly affected. However, at the balloon surface, attenuation on the order of 18.0% is observed. The AAPM TG‐43 source data currently incorporated in commercially‐available treatment planning systems do not account for the variable dose distributions attributable to balloon wall attenuation. Our results show that variable attenuation factors that may have clinical significance should be applied in order to determine near‐surface dose distributions when using barium impregnated balloons for intracavitary breast brachytherapy. Dose distributions at distances greater than 1 cm from the surface of the balloon appear to be accurately represented without further modification.

PACS numbers: 87.53.Jw, 87.55.D‐, 87.55.d, 87.55.Qr, 87.55.K‐

## I. INTRODUCTION

Xoft, Inc., (Sunnyvale, CA), recently introduced a technique for administering accelerated partial breast irradiation (APBI)^(1)^ by electronic brachytherapy (EBT) that employs a low energy (50 kV) electronic radiation source administered through a balloon applicator implanted in the tumor cavity and inflated with saline solution. To deliver a prescribed radiation dose, the system controller moves the radiation source through the balloon catheter, stopping at a series of dwell positions for predetermined times. These dwell positions and times are computed with a commercially‐available brachytherapy treatment planning system and transferred to the controller.

The treatment planning methodology used in EBT is similar to that of high dose radiotherapy (HDR) with Iridium‐192. For EBT, the manufacturer provides specialized TG‐43 parameters^(^ ^2^ ^,^ ^3^ ^)^ including active source length, air kerma strength, dose rate constant, radial dose function and anisotropy function.

**Figure 1:**
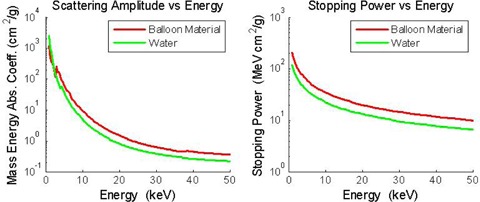
Scattering amplitude and stopping power versus energy for the barium impregnated balloon material compared to water.

In order to facilitate the treatment of different tumor bed shapes and sizes, balloon applicators are available in sizes ranging from 4 to 6 cm, spherical and 5 by 6 cm and 5 by 7 cm, ellipsoidal. The balloon portion of the applicator is made from a proprietary silicone‐based material impregnated with barium sulfate. The barium is introduced into the balloon material to allow the balloon to fluoresce during imaging and treatment simulation. However, during a treatment, the barium absorbs some of the ionizing radiation to a greater degree than would an equivalent thickness of water, thereby attenuating the planned patient dose. To demonstrate this phenomenon: Fig. 1 is a comparison of the scattering amplitudes for photons and the stopping power for electrons for the barium impregnated balloon material and water.^(^ ^4^ ^,^ ^5^ ^)^ The barium impregnated balloon material has a greater scattering amplitude and stopping power than does an equivalent amount of water, suggesting that more energy will be absorbed by the balloon material than would be absorbed by the same thickness of water in the energy range of the source spectrum, thus restricting the passage of ionizing radiation through the balloon wall to the patient.

Since the treatment planning system utilizes a homogenous medium dose calculation and does not consider attenuating material in the radiation path, resulting computed doses will be inaccurate unless appropriately modified. The manufacturer accounts for the balloon wall attenuation by applying a universal correction factor and increasing all computed dwell times by 6.0%. This factor was derived by measuring the dose at 1.0 cm from the balloon surface in a water phantom in the presence and absence of the silicon material and comparing the two results. A single correction factor, however, does not account for all variables that influence the dose distribution. For example, the thickness of the balloon material at different polar angles will play a large role in determining the attenuation due to differences in scattering amplitude and stopping power.

This study was conducted to gain additional knowledge about the true dose attenuation of low‐energy radiation that passes through various thicknesses of barium‐impregnated balloon material before entering the patient's tissues.

## II. MATERIALS AND METHODS

Numerical and experimental analyses were conducted to determine depth dose curves for various balloon wall thicknesses with and without barium impregnation. The extent of the experimental investigation was limited to a subset of all the data accumulated as a verification of the numerical accuracy.

It was determined that when the balloon is maximally inflated, its thickness at the apex, normal to the central catheter, is approximately 0.3 mm and thickens to approximately 0.55 mm at the distal and proximal ends. Therefore, the amount of balloon material an X‐ray will pass through is assumed to be between 0.3 mm and 0.6 mm. Experimental data for thicknesses of 0.4 mm and 0.5 mm were obtained to verify the numerical results for the full span of thicknesses needed for the analysis.

### A. Experimental measurements

A plane parallel plate extrapolation ionization chamber, (Model EIC‐1, Far West Technology, Inc., Goleta, CA), was used to determine dose in the buildup region near the surface.^(6)^ Measurements were taken with 1.3, 2.3, 3.3, and 4.3 mm electrode separations and the surface dose estimated by extrapolating these readings to a zero mm electrode separation. The bias voltage was adjusted to maintain a 50V/mm potential between the electrodes. Two dose readings with opposite bias polarity were obtained and the results averaged.

**Figure 2:**
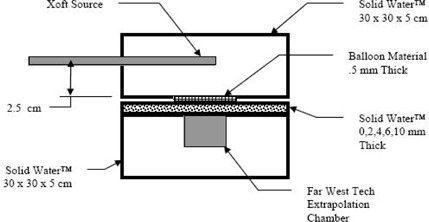
Experimental setup showing the placement of the EBT X‐Ray source, balloon material, and the extrapolation chamber.

The experimental setup simulated an EBT X‐ray source positioned 2.5 cm from the balloon's outer wall with wall thicknesses of 0.4 mm and 0.5 mm. The extrapolation chamber was set at 0, 2.0, 4.0, 6.0, and 10.0 mm distant from the wall to obtain depth dose readings. The balloon material was stretched to the desired thickness and fastened to a 30 cm×30 cm×5 cm thick Solid Water phantom (Gammex, Middleton, WI). The extrapolation chamber was placed in an additional 30 cm×30 cm×5 cm thick phantom. Phantom thicknesses of 0, 2.0, 4.0, 6.0, and 10.0 mm were positioned between the two 5.0 cm slabs, as pictured in Fig. 2 and measurements obtained for each slab thickness.

To understand the effects of the interposed balloon material on the dose distribution, a second set of readings was taken with a water equivalent material of the same thickness, positioned in place of the balloon material with no dimensional changes to the setup shown in Fig. 2.

Finally, a check with a balloon thickness of 0.4 mm was performed with readings taken at depths of 2.0 mm and 10.0 mm.

### B. Numerical analysis

To perform the numerical calculations, a general purpose multi‐particle transport code (FLUKA) was used.^(7)^ FLUKA, which is supported by the European Organization for Nuclear Research (CERN), is a numerical tool for the calculation of particle transport and interactions with matter. It is designed for applications that include shielding, calorimetry, dosimetry, detector design and radiotherapy.

**Figure 3:**
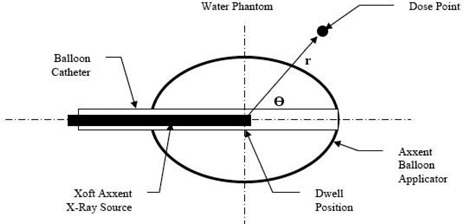
Numerical model used for the analysis of the balloon material. The X‐ray source is shown in the central dwell position but may be positioned at any of the possible dwell positions. The dose point is referenced by specifying the radial position, r, and polar angle, Θ, from the dwell position.

Figure 3 shows a typical arrangement of the EBT source and balloon. However, source position and the shape of the balloon are variable.

**Figure 4:**
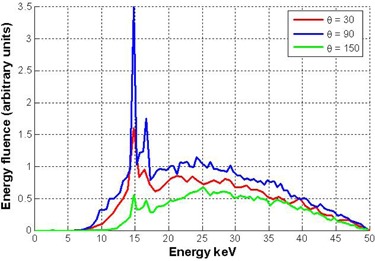
Sample of the energy spectrum calculated using FLUKA for the numerical analysis. The spectra are normalized at xTH=90° and energy=23 keV.

To proceed with the numerical analysis, the energy spectrum of the EBT source was determined at various polar angles, Θ. To accomplish this, an accurate model of the source was constructed and the energy spectrum at angles ranging from 0° to 180° in 5° increments was computed using FLUKA. Examples of the spectra for Θ=30°,90°, and 150° are shown in Fig. 4. The energy spectra for the 36 angles (not all shown) agreed well with the manufacturer's reported spectra.^(8)^

**Figure 5:**
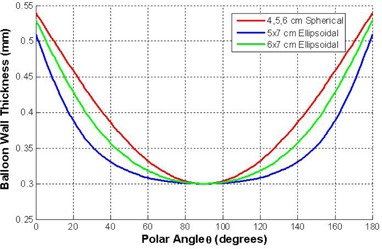
Plot of the functions to determine the thickness of the applicator balloon material as a function of polar angle Θ normal to the surface.

As a consequence of using a low energy source, the thickness of the balloon material traversed by the photons greatly affects the amount of radiation absorbed by the balloon. Therefore, the thickness of the balloon material as a function of the polar angle, Θ was determined. This information was obtained by observing that the thickness of the balloon is approximately proportional to the hoop stress of the inflated balloon at that angle and is also closely proportional to the cosine of the slope of the surface. Using this methodology, the thickness of the balloon as a function of polar angle can be approximated (Fig. 5). In addition, the source dwell position plays a significant role in determining the amount of balloon material through which photons must travel, due to the angle of incidence between the radiation direction and the balloon surface. This is particularly true when the dwell position is close to the distal or proximal ends of the balloon.

Several FLUKA simulations were performed at various balloon wall thicknesses, and dose as a function of distance from the balloon wall recorded with and without barium impregnation.

## III. RESULTS

**Figure 6:**
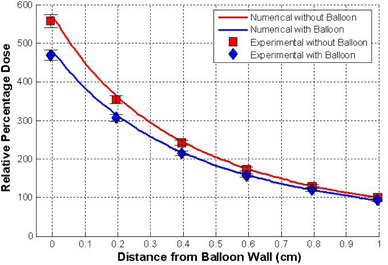
Comparison of the experimental results and the numerical results for balloon wall thickness of 0.5 mm plotted as a function of distance from the balloon wall.

To confirm the accuracy of the numerical model, the setup of the experimental analysis was duplicated numerically and the two results compared. Figure 6 shows the percent depth dose curves as measured experimentally and computed numerically for a balloon wall thickness of 0.5 mm, with and without the balloon applicator material. The depth dose curves were normalized to 1.0 cm for the case without the balloon material. The numerical results are within the error bars of the experimental results. This was also true for the case when the balloon material was stretched to a thickness of 0.4 mm.

**Figure 7:**
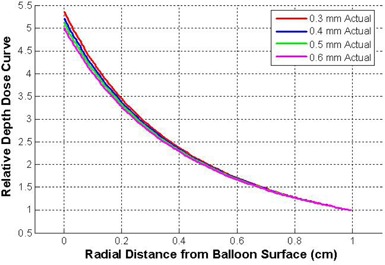
Depth dose curves for balloon thicknesses 0.3, 0.4, 0.5, 0.6 mm. The curves are normalized at the 0.3 mm thickness at a distance of 1 cm.

The depth dose curves for balloon thicknesses 0.3, 0.4, 0.5, and 0.6 mm were computed numerically with the results shown in Fig. 7. All depth dose curves are normalized to the 0.3 mm thick curve at 1 cm from the balloon surface.

**Figure 8:**
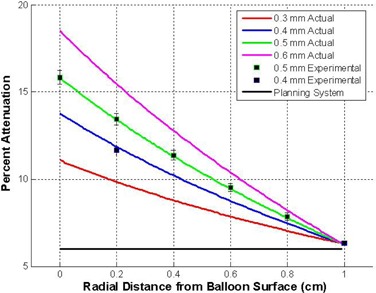
Attenuation curves for various balloon thicknesses. These curves represent the percent difference of the dose depth curve from the barium impregnated balloon and an equivalent thickness of water.

Attenuation of the ionizing radiation as a function of balloon thickness is presented in Fig. 8. These curves represent the percent attenuation of the photons as they pass through a layer of balloon material relative to their passage through a water layer of the same thickness. From the graphs, it can be seen that a nominal attenuation of 6.0% is found at the normalization point of 1.0 cm, as predicted by the manufacturer. However, the attenuation is directly proportional to the balloon wall thickness; for a 0.6 mm thick balloon, the attenuation increases to 18%.

Figure 8 also shows the accuracy of the numerical analysis for both the 0.4 mm and 0.5 mm thicknesses, as compared to the experimental results.

## IV. DISCUSSION

Dose depth curves were computed for a balloon applicator for various balloon wall thicknesses with and without barium impregnation of the balloon material. From this data, attenuation curves were computed for each of the balloon thicknesses investigated. The analysis shows a consistent attenuation of 6.0 percent for all thicknesses at the 1.0 cm prescription point, as predicted by the manufacturer. The analysis also reveals a greater attenuation of the X‐ray energy between the balloon surface and the 1.0 cm prescription point for all wall thicknesses. Referring to Fig. 8, an X‐ray that passes through 0.6 mm of barium impregnated balloon material will demonstrate an attenuation of 18% at the balloon surface, which is a factor of three times greater than that assumed by the manufacturer. This might have significant clinical implications on cell survival of malignant cells close to the balloon surface. Commercial treatment planning systems do not correct for this attenuation and, therefore, report a higher dose to the patient in this region than actually is delivered which, in turn, implies less cell kill.

**Figure 9:**
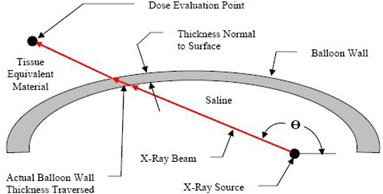
Geometry section showing the actual thickness of balloon material an X‐ray traverses on the path to a dose point.

For this analysis, a maximum balloon wall thickness of 0.6 mm was used based on dimensions normal to the surface. There are cases, however, where the thickness of balloon material will be larger than 0.6 mm if the incident angle of the X‐ray with the balloon surface is taken into account. This is evident from Fig. 9 where two different X‐ray paths lead to greater distance traveled by an X‐ray through the balloon material.

The ability of currently available treatment planning systems to account for the attenuation of the energy in barium impregnated balloon material is limited by the computational model specified in the AAPM TG‐43 specification. The TG‐43 anisotropy function $F_{L}(r,\Theta )$, and the radial dose function $g_{L}(r)$ could be modified to account for the barium attenuation if only a single source dwell position is considered for a particular balloon shape. However, the wide variety of balloon applicator shapes and sizes, as well as the number of possible dwell positions specified in a typical treatment plan, make the task challenging for actual source configurations to be used in treatment.

## V. CONCLUSIONS

The attenuation curves computed in this analysis are in agreement with a universal correction factor only at the 1.0 cm prescription point. However, there is a significant difference close to the surface of the balloon. This was confirmed using a parallel plate extrapolation ionization chamber to accurately measure surface dose. Given this dose differential, the TG‐43 anisotropy function $F_{L}(r,\Theta )$ and the radial dose function $g_{L}(r)$ that are at present routinely applied, cannot accurately account for the variations in applicator size and shape and source dwell position. Furthermore, a planning system using the TG‐43 formulization without modification will not compute the dose distribution accurately when attenuating material, such as a barium impregnated balloon applicator wall, is in the path of the ionizing radiation.
